# Analysis of Ionospheric Scintillation Characteristics in Sub-Antarctica Region with GNSS Data at Macquarie Island

**DOI:** 10.3390/s17010137

**Published:** 2017-01-12

**Authors:** Kai Guo, Yang Liu, Yan Zhao, Jinling Wang

**Affiliations:** 1 School of Instrumentation Science and Opto-electronics Engineering, Beihang University, Beijing 100191, China; k_guo@buaa.edu.cn (K.G.); Zhaoyan@buaa.edu.cn (Y.Z.); 2 Collaborative Innovation Center of Geospatial Technology, Wuhan 430079, China; 3 School of Civil and Environmental Engineering, University of New South Wales, Sydney, NSW 2052, Australia; jinling.wang@unsw.edu.au

**Keywords:** ionospheric scintillation, sub-Antarctica region, temporal and spatial characteristics, GNSS signals

## Abstract

Ionospheric scintillation has a great impact on radio propagation and electronic system performance, thus is extensively studied currently. The influence of scintillation on Global Navigation Satellite System (GNSS) is particularly evident, making GNSS an effective medium to study characteristics of scintillation. Ionospheric scintillation varies greatly in relation with temporal and spatial distribution. In this paper, both temporal and spatial characteristics of scintillation are investigated based on Macquarie Island’s GNSS scintillation data collected from 2011 to 2015. Experiments demonstrate that occurrence rates of amplitude scintillation have a close relationship with solar activity, while phase scintillation is more likely to be generated by geomagnetic activity. In addition, scintillation distribution behaviors related to elevation and azimuth angles are statistically analyzed for both amplitude and phase scintillation. The proposed work is valuable for a deeper understanding of theoretical mechanisms of ionospheric scintillation in this region, and provides a reference for GNSS applications in certain regions around sub-Antarctica.

## 1. Introduction

Ionosphere is a layer in the earth’s upper atmosphere from 60 to 600 km. Under the irradiation of the sun, molecules in this region can be decomposed into ions and electrons, which results in an interference for the signals passing through this region [1]. The disturbed signals might suffer a sharp attenuation in amplitude and random variations in phase. This is referred as ionospheric scintillation. Scintillation has attracted extensive researches in recent years due to its considerable influence on electromagnetic signals.

The major impact of ionospheric scintillation on GNSS (Global Navigation Satellite System) can be roughly divided into two categories [2,3,4,5,6]. Firstly, it probably causes a severe decrease in amplitude leading to cycle slips, or even a complete loss of lock to satellite signals for GNSS receivers. Secondly, the rapid fluctuation on phase may result in an increase of tracking loop errors. The threat caused by ionospheric scintillation is non-ignorable. However, the distorted signals conversely provide a medium to study and model the ionosphere. Some modified GNSS receivers were therefore employed to observe the event of scintillation, such as ionospheric scintillation monitors (ISMs) [7], etc.

Extensive studies have been carried out on ionospheric scintillation. The severest ionospheric scintillation usually occurs at low latitudes because of the effect of Rayleigh–Taylor instability around equatorial region. Some sectors around Eastern Asia have been widely focused on and studied, taking the geomagnetic storm influence into account [8]. It is widely accepted that scintillation is closely related to the behavior of plasma bubbles, which has been deeply investigated during storm time at middle latitudes [9]. This study sheds light on particularities of regional characteristics of scintillation and its internal physical mechanism may be determined by several factors comprehensively. Researchers [10] have studied the scintillation characteristics and scintillation effects on Global Positioning System (GPS), based on the data sets collected in July and August 2012 in Hong Kong. The influence of scintillation on GPS signals has also been studied in European Arctic from 8 to 14 November 2004 [11]. In addition, researchers at Stanford University investigated aviation GNSS performance under ionospheric scintillation [2]. However, those previous studies were implemented with a limited number of observations, and some focus too much on the data during solar maximum or geomagnetic storms. For instance, influence of scintillation on GNSS signals was discussed in Brazil in the 2012 to 2013 solar maximum [12]. To better understand and model scintillation, more comprehensive studies are required based on more complete and overall scintillation data sets.

To address the problems above, in this paper the temporal and spatial behaviors of scintillation observed at Macquarie Island is investigated in detail based on the data sets provided by Space Weather Service (SWS) of Australia. The observation time span is as long as five years from 2011 to 2015. The observatory is one of the space weather monitor stations in sub-Antarctica region and data sets processed in this paper were collected and selected by strict criteria to form a reliable data pool for further investigations. Based on the numerous data sets, this work aims at contributing to a better understanding of the scintillation characteristics in sub-Antarctica region. To accomplish this task, temporal distribution features are firstly investigated. A general picture of the amplitude and phase scintillation of different intensities is displayed to illustrate the seasonal variation property. Then, monthly and daily scintillation occurrence rates are calculated and plotted with Sunspot Number (*SSN*), verifying that solar activity considerably influences monthly scintillation of amplitudes. The correlation between the geomagnetic field activity and the ionospheric scintillation is also discussed in this paper. It is found that the daily phase scintillation occurrence rate maintains a strong relationship with geomagnetic index *Ap*, *Ae* and geomagnetic field *H* component. Finally, the statistical spatial distributions of scintillation are discussed. The results show that the phase scintillation mainly focuses on two invariable regions during all the time spans. However, for the amplitude scintillation, the active region changes with the year.

The rest of the paper is organized as follows. In Section 2, a further description of ionospheric scintillation is provided as a baseline theory for the proposed study. The method of calculating correlation coefficient is also discussed in this section. The proposed methods for data collection and preprocessing are also involved. In Section 3, experiments are set up and the results are discussed thoroughly based on the data preprocessed in Section 2. The conclusions and summaries of this study are given in Section 4.

## 2. Material and Methods

### 2.1. Basic Theory of Ionospheric Scintillation

When a radio frequency signal transmits through the ionospheric irregularities, the amplitude and phase are likely to suffer an intense fluctuation, called ionospheric scintillation [13,14]. Typically, the ionospheric scintillation can be classified into amplitude scintillation and phase scintillation. The widely used indices to represent these two kinds of scintillation are *S*_4_ and *σ_φ_* separately. Amplitude scintillation index *S*_4_ signifies the standard deviation of normalized signal power, given by:

(1) $$S_{4}=\sqrt{\frac{\langle I^{2}\rangle -{\langle I\rangle }^{2}}{{\langle I\rangle }^{2}}},$$

where *I* is the intensity (power) of the signal and is computed by *I = A^2^*; *A* denotes the amplitude of the signal; and <•> indicates the average during a certain time interval. In this paper, *S*_4_ is calculated every 60 s. The *S*_4_ index can be directly calculated from GPS signal intensity [10,15]. Usually, it falls into the range of 0 to 1.

Phase scintillation index *σ_φ_* is defined as the standard deviation of normalized signal phase, given by:

(2) $$\sigma_{\varphi }=\sqrt{\langle \phi^{2}\rangle -{\langle \phi \rangle }^{2}},$$

where *φ* is the de-trended phase of the signal [15,16]. *σ_φ_* cannot be directly calculated from GPS data, and it is obtained from de-trended phase observations.

Previous researches have shown that occurrence of ionospheric scintillation is directly correlative with the inhomogeneity and irregularity of ion clusters, the shift of ionospheric plasmas etc. [17]. Moreover, properties of ionosphere are affected by many factors, e.g., solar and geomagnetic activities, the local time etc. According to [18], there exists a strong dependence on the solar activity for occurrence rates and intensities of ionospheric scintillation. Furthermore, with the change of the relative position between the earth and the sun, scintillation presents a seasonal variation with peaks on the equinox months, which has been widely studied and verified [19,20].

Ionospheric scintillation is also considered to be relevant to the location of observatory, and scintillation is more serious in equatorial and polar areas than that at middle latitudes [21]. Additionally, the phase scintillation is more active and severer than the amplitude one in high latitude areas [22,23]; while in low latitude areas, the amplitude scintillation is more considerable [24,25]. Researchers suggested that ionospheric scintillation is directly related with disturbance of geomagnetic field. The generally believed reason is that the geomagnetic disturbance induces more electric and radio irregularities in the surface of the earth [26].

To quantify the degree of the correlation between scintillation and solar or geomagnetic field activities, correlation coefficients are introduced in this paper. This index reflects the level of correlation between two variables and it is given by:

(3) $$R_{X,Y}=\frac{\mathrm{cov}\left(X,Y\right)}{\sqrt{\mathrm{var}\left(X\right)\times \mathrm{var}\left(Y\right)}},$$

where *X* and *Y* are sample vectors of scintillation and solar or geomagnetic field activities, respectively. The correlation coefficient is a dimensionless quantity and the absolute value will not surpass 1. In addition, the closer the indicator is to 1, the more related the variables are.

### 2.2. Database

The GPS scintillation data sets processed in this paper were collected from World Data Center (WDC), which is organized by SWS. Scintillation data handled in this paper were measured by observation station Macquarie Island (geographic: 54.50° S, 158.95° E; geomagnetic: 64.54° S, 248.10° E), which is one of the Ionospheric Prediction Services Network (IPSNET) space weather monitoring stations in sub-Antarctica located within the 90% auroral zone [27]. The numbers of equivalent days with scintillation data available are listed in Table 1.

It should be noted that GPS Silicon Valley ISMs were employed by SWS for observation at this monitor station. These ISMs were built based on the dedicated Novatel OEM GPS receiver cards. Therefore, these receivers can maintain locked to satellites even under strong scintillation.

The scintillation data processed were recorded every 1 min in daily data files which contain nine basic parameters, as shown in Table 2. Unfortunately, phase scintillation index is not available in the data files until May 2012. Thus, in the proposed work, only *S*_4_ index was processed before that date.

In this paper, Final_*S*_4_ and Sigma60, also known as *S*_4_ and *σ_φ_*, respectively, are adopted to indicate the intensity of amplitude and phase scintillation. Based on the two indices extracted from the data files, scintillation intensity is divided into four levels as shown in Table 3.

It is worth mentioning that in this research only the satellites with ray paths above 30° elevation were considered aiming to minimize the impact of multipath effects. With the multipath interference considerably removed, the scintillation characteristics can be better explored.

### 2.3. Analysis Strategy

**Figure 1:**
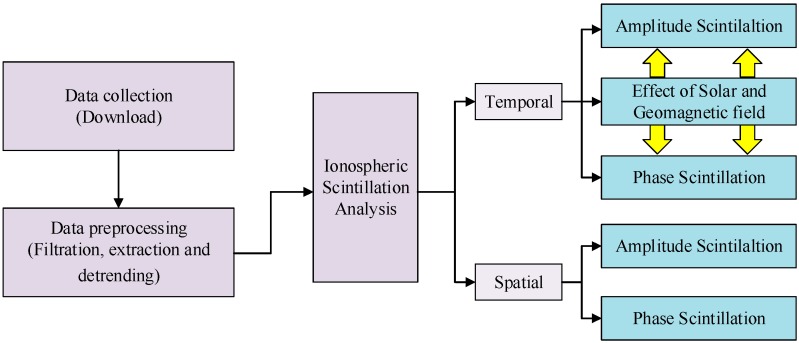
The overall process of data analysis.

The focus of this research is to statistically analyze the characteristics of ionospheric scintillation observed at Macquarie Island. The overall process of data analysis is demonstrated in Figure 1. According to the figure, one of the key points is the analysis of temporal characteristics of the scintillation. Based on the data collecting and preprocessing process, influences of solar and geomagnetic field activities on amplitude and phase scintillation are deeply discussed, respectively. Another key point is the investigation of the spatial behaviors of the scintillation. Spatial distributions of both amplitude and phase scintillation measured at this station are discussed in detail. In addition, a Graphical User Interface (GUI) is also developed in the overall process to make the research more convenient.

## 3. Experiments

### 3.1. General Temporal Statistics of Ionospheric Scintillation

**Figure 2:**
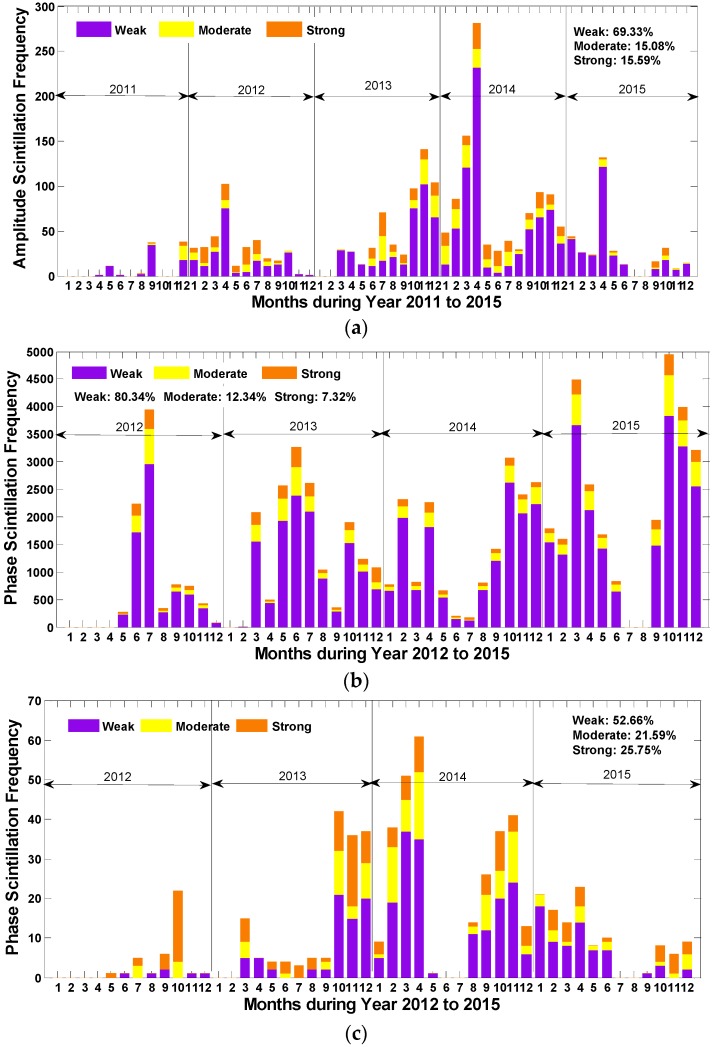
(**a**) Statistics of amplitude scintillation; (**b**) statistics of phase scintillation; and (**c**) statistics of phase scintillation on the condition that amplitude scintillation takes place.

In this section, an overall temporal statistical distribution for amplitude and phase scintillation of different intensities observed during the 5 years (4 years for phase scintillation) was first investigated separately, as shown in Figure 2. According to Figure 2a, the amplitude scintillation demonstrates an apparent seasonal variation, reaching peaks in spring and autumn and bottoming out in summer and winter. By contrast, there is no sensible seasonal fluctuation for phase scintillation, though the difference between the months is also manifest as illustrated in Figure 2b. Instead, the phase scintillation frequency is likely to peak in summer and winter in most years.

It can also be seen that the weak scintillation accounts for considerable proportions for most months, which mainly contributes to the frequency differences between months for both amplitude and phase scintillation. In addition, the appearance of phase scintillation is more frequent than that of amplitude scintillation, which is consistent with the most recent study that concluded phase scintillation is more active in high latitude regions [22].

Figure 2c presents the frequency of phase scintillation on the condition that amplitude scintillation takes place. It can be seen that the number of amplitude and phase scintillation occurring at the same moment varies largely among different months. The total number of the scintillation in Figure 2c accounts for 26.37% of that in Figure 2a and 0.86% in Figure 2b during 2012 to 2015, which means that the phase scintillation probably appears alone and approximately one quarter of the amplitude scintillation takes place together with phase scintillation. Furthermore, phase scintillation of moderate and strong levels accounts for a larger proportion when both kinds of scintillation occur, according to the percentages marked in the figures.

### 3.2. Solar Activity and Ionospheric Scintillation

Monthly scintillating occurrence rates were calculated to investigate the relationship between solar activity and ionospheric scintillation in this research. The scintillation occurrence rate is defined as the ratio of the number of scintillation occurred (*S*_4_ ≥ 0.2 or Sigma60 ≥ 0.2) to the total scintillation samples during a certain period of time, which can be a month, a day, an hour, etc. The scintillation occurrence rate can reflect the frequency of scintillation activity properly and it has been widely adopted in scintillation researches.

**Figure 3:**
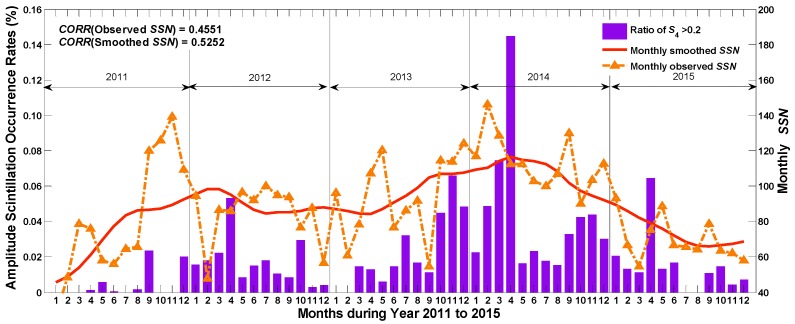
Amplitude scintillation occurrence rate (per month) and *SSN*.

As shown in Figure 3, the occurrence rates of amplitude scintillation are positively correlated with the variation of the monthly *SSN*, especially with the monthly smoothed *SSN*, which is calculated by a running mean of the monthly *SSN* over 13 months. To quantify the level of correlation, the correlation coefficients are calculated and labeled in the figure. The correlation coefficient between the occurrence rates and the monthly smoothed *SSN* is 0.53, which is higher than monthly observed *SSN*. Additionally, it is shown that the occurrence rate in April 2014 was extremely high. This outstanding value is coincidentally consistent with the maximum smoothed *SSN*, which peaks in the same month. Further research might be conducted to analyze the reasons for the extremely strong amplitude scintillation in that month.

**Figure 4:**
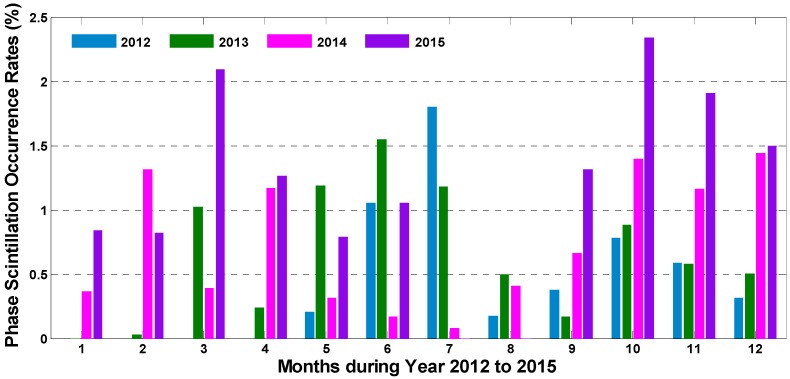
Monthly phase scintillation occurrence rates.

Compared with the amplitude scintillation, the phase scintillation is not strongly correlated with *SSN*. Figure 4 unfolds the monthly occurrence rates for phase scintillation during the researched years. It can be seen that the occurrence rates probably peak in March, July, October and November. The overall frequency of phase scintillation in 2015, especially in the latter half of the year, is much higher than that in other years. In addition, there is an obviously gradual increase in September, October, November and December among different years, which is really against the regularity of solar activity. This gradual change might be associated with the variation of the earth’s physical environment. Thus, more investigations should be implemented in a separate study for further explanation of this unreasonable tendency.

To analyze the impact of solar activity on ionospheric scintillation, the correlation coefficients between *SSN* and daily scintillation rates were also computed. It should be mentioned that the number of scintillations appearing during one day can be extremely low, which probably causes misleading statistical results. Thus, only data sets from eight months were selected to calculate the daily scintillation rates in this paper. These months were chosen from 2012 to 2015 and each month has a higher occurrence rate, either for amplitude scintillation or for phase scintillation, in the corresponding year. Data from 2011 were not included since phase scintillation data were not available in this year. These selected months are listed in Table 4.

**Figure 5:**
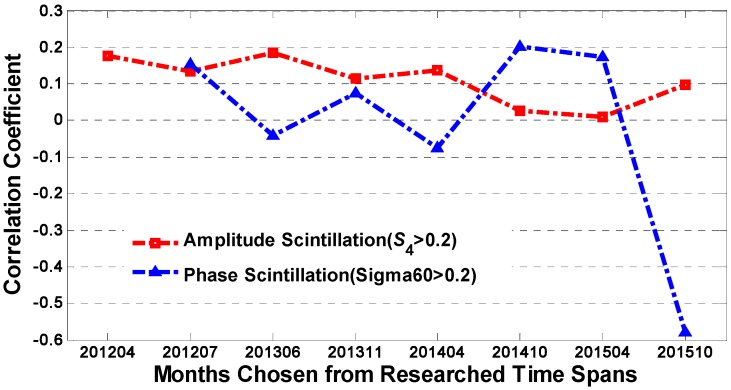
The correlation coefficients between the daily scintillation rates and the monthly smoothed *SSN*.

The correlation coefficients between daily scintillation rates and monthly smoothed *SSN* are demonstrated in Figure 5. Most of the output values fall into the range of −0.1 to +0.2, indicating that there is little relationship between *SSN* and the daily occurrence rates for both amplitude and phase scintillation. This result does not agree with the former conclusions obtained from Figure 3 that the monthly occurrence ratios of amplitude scintillation are primarily impacted by solar activity. It is supposed that this unusual result can be explained as follows: the effect of solar activity on scintillation is more reflected as a kind of indirect cumulative effect, especially for the amplitude scintillation. Meanwhile, the amplitude scintillation appears rarely around high latitude regions. It is more like a random event that cannot present obvious variation patterns. For phase scintillation, solar activity might have even less impact. However, the correlation coefficient in October 2015 is anomalous reaching −0.6. It is unusual and deserves a further analysis.

### 3.3. Geomagnetic Field Activity (Ap) and Ionospheric Scintillation

**Figure 6:**
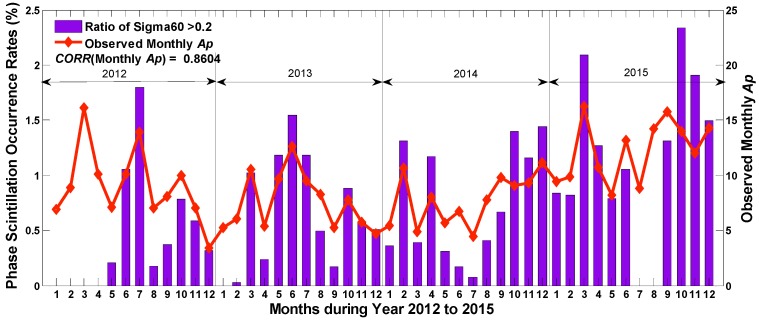
The monthly phase scintillation rates against the variation of monthly *Ap*.

Ionospheric scintillation can also be affected by the geomagnetic field activity, which can be globally indicated by *Ap* and *Kp*. As *Ap* index is converted from *Kp* value linearly, only *Ap* is employed here. Another indicator adopted to demonstrate geomagnetic storms in this paper is Auroral Electrojet (*Ae*) index, which mainly quantifies the magnetic activity in auroral zone. In this section, the correlation values between monthly occurrence rates of scintillation and *Ap* or *Ae* were first calculated. It is found that the phase scintillation is positively correlated with both *Ap* and *Ae* with high correlation coefficients of 0.86 and 0.74, respectively. For amplitude scintillation, the respective correlation coefficients are much lower, only 0.11 and 0.13. Figure 6 illustrates the monthly phase scintillation rates against the variation of monthly *Ap*. It can be seen from the figure that the occurrence of phase scintillation varies apparently along with the geomagnetic indicator *Ap*.

**Figure 7:**
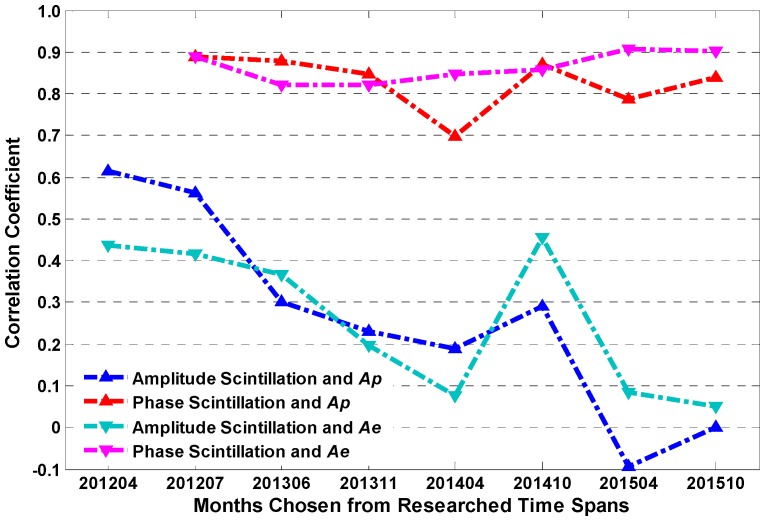
The correlation between scintillation occurrence rates and *Ap* and *Ae*.

Similar to Section 3.2, the correlation coefficients between daily scintillation occurrence rates and *Ap* and *Ae* are calculated. Again, only scintillation presented in the months listed in Table 4 was counted. Figure 7 illustrates the correlation between geomagnetic activity index and amplitude and phase scintillation occurrence rates. As we can see in the figure, most of the correlation values for amplitude scintillation remain relatively low over all of the studied months and exhibit an apparent decline from 2012 to 2015, indicating that the degree of correlation between *Ap*, *Ae* and amplitude scintillation decreases gradually year by year. By contrast, the phase scintillation is highly correlated with *Ap* and *Ae*. The correlation coefficient remains high over most researched months, especially for *Ae*, which even exceeds 0.9 in April 2015.

**Figure 8:**
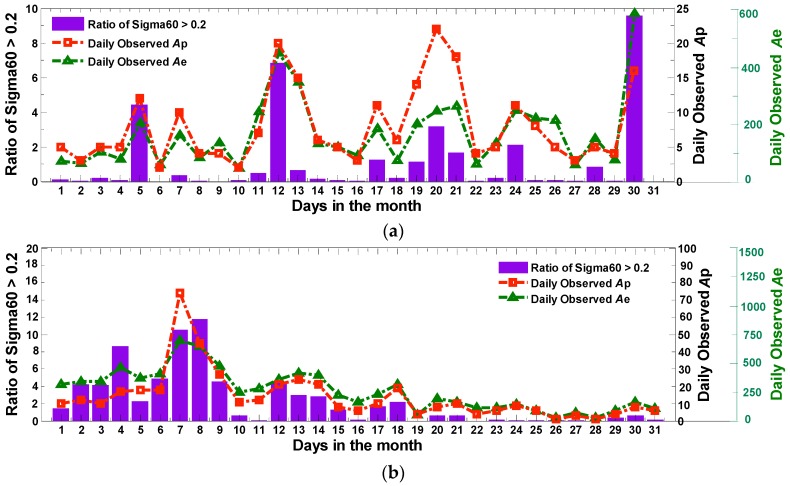
The variation of daily phase scintillation rates against *Ap* and *Ae* in: April 2014 (**a**); and October 2015 (**b**).

Based on the research above, it can be concluded that the geomagnetic field activity has a stronger influence on the daily amplitude and phase scintillation. Compared with solar activity, the geomagnetic field activity can directly impact the daily occurrence of scintillation, especially for the phase scintillation. Thus, the geomagnetic field probably acts as one of the main contributory factors to scintillation. As an example, Figure 8 displays the variation of daily phase scintillation rates against *Ap* and *Ae* in April 2014 and October 2015.

Meanwhile, it can also be found that the correlation between the geomagnetic activity and scintillation varies in different months, which indicates that there might be some other factors affecting the ionospheric scintillation in this area. Considering the focus of this paper is to present a general understanding of the scintillation at Macquarie Island, we will not go into details of the result. Further analyses about this special result will be conducted in the future work.

### 3.4. Geomagnetic Field Activity (H) and Ionospheric Scintillation

As it has been previously studied, the daily scintillation occurrence rates, especially for phase scintillation, are closely modulated by geomagnetic field index *Ap* and *Ae*. However, *Ap* index is obtained from 13 magnetic observatories at mid-latitudes. The indicator therefore cannot properly represent the level of geomagnetic activity at Macquarie Island, which is located near high latitudes. Additionally, *Ap* is calculated every three hours, and cannot reflect the specific changes of the geomagnetic field. Similarly, the value for *Ae* index is measured by several monitor stations along the northern auroral zone, which is not an accurate description for the geomagnetic field activity at Macquarie Island either. Consequently, horizontal component *H* of the geomagnetic field measured by the magnetic observatory at Macquarie Island is adopted here to further investigate the relevance between daily scintillation occurrence rates and the geomagnetic field.

**Figure 9:**
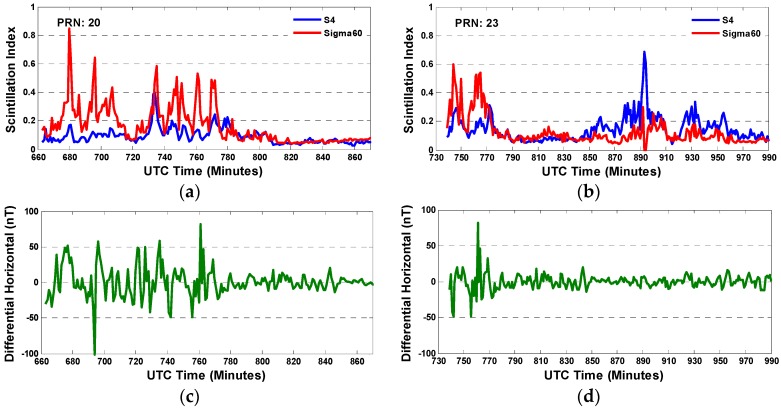
The variation of scintillating indices of PRN 20 (**a**); and PRN 23 (**b**); (**c**,**d**) are the differential *H* component of the geomagnetic field during the corresponding time spans.

In this experiment, the scintillation data on April 5th 2014 was processed to reveal the variation of scintillation index against *H* component, as shown in Figure 9. The amplitude and phase scintillation indices for PRN 20 and 23 are plotted in the first line of the figure respectively. In the second line, the differential *H* component is presented corresponding to the researching time span in the first line. By comparing the sub-figures by column, it can be found that the intensity of scintillation is related to the strength of the differential *H*, which indicates the *H* fluctuation properly. A drastic change of *H* component produces a strong and undulated scintillation. Besides, for PRN 23, the considerable amplitude scintillation occurs from minute about 875 to 895, while the geomagnetic field is relative quite at that time, indicating that there might be other factors affecting the scintillation. More studies might be conducted to further investigate the principles for that.

**Figure 10:**
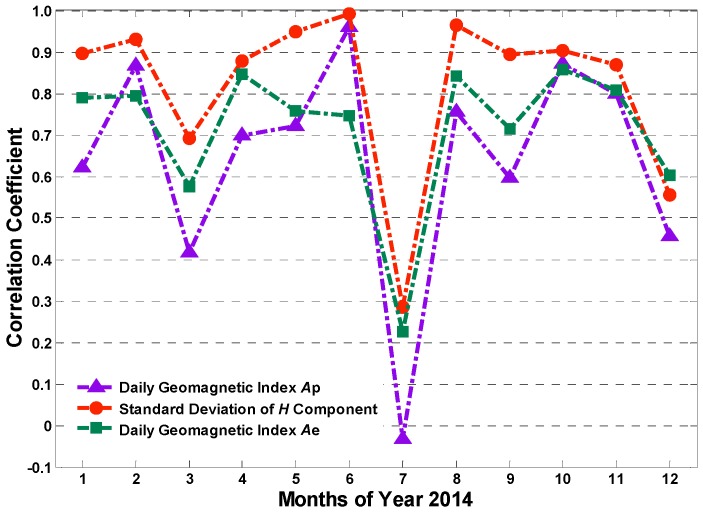
The comparison between the correlation coefficients for daily *Ap* and *Ae*, the standard deviation of *H* component and the monthly occurrence rates of phase scintillation.

To further compare the effects of geomagnetic field index and *horizontal component* *H* on the occurrence rates of phase scintillation, another analysis is implemented, as shown in Figure 10. The red dot dash line in Figure 10 demonstrates the variation of correlation coefficients between the daily phase scintillation occurrence rates and the standard deviation of geomagnetic field *H* *component* for every month in 2014, while the blue and green dot dash lines correspond to observed daily geomagnetic index *Ap* and *Ae*, respectively. It is obvious that the red dots are higher than the other ones over all the months, distinctly indicating that the correlation degree for the standard deviation of *H* *component* is stronger than that of geomagnetic indices. Besides, the correlation coefficients in July for all lines are notably lower than other months, which needs further research in future. Based on this analysis, it can be concluded that the phase scintillation depends more on the local geomagnetic field component *H*. The conclusion is reasonable and of great significance for scintillation prediction.

### 3.5. Spatial Distribution of Scintillation

Above studies chiefly focus on the temporal distribution characteristics of the scintillation observed at Macquarie Island during 2011 to 2015. In this subsection, we concentrate on the spatial distribution characteristics of scintillation. The geomagnetic field disturbance has a strong influence on scintillation index; however, it is interesting to note that not all the satellites will encounter scintillation at the same moment. Besides, the intensities of scintillation for different satellites also vary largely. All those cases indicate that the intensity and frequency of scintillation are associated with the different satellite positions in orbits in the sky.

In this experiment, all amplitude and phase scintillation indices are yearly analyzed according to the elevation and azimuth angles. On the premise that scintillation appears, the frequency of scintillation appearing in different regions are calculated and plotted in the sky charts by year. The charts are unfolded from a sky view, in which the center is the observatory local zenith and the circles around it are equal elevation contours. The lower bound of the elevation in the process is set to 30° to minimize multipath effects, therefore no scintillation appears outside the 30° elevation contours.

**Figure 11:**
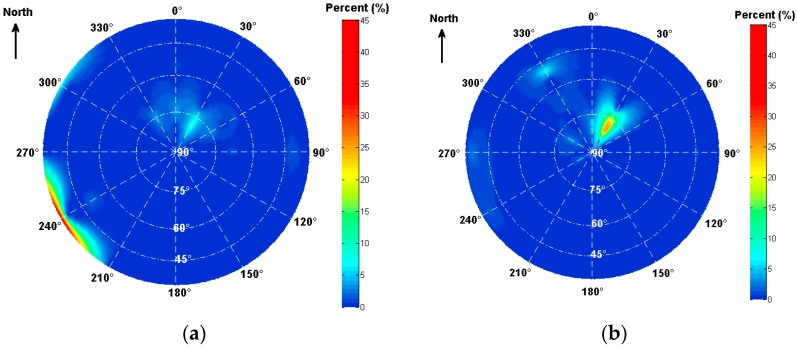
The distribution of frequency for amplitude scintillation observed in: 2012 (**a**); and 2015 (**b**).

Figure 11 illustrates the distribution of amplitude scintillation observed in 2012 (Figure 11a) and 2015 (Figure 11b). As can be seen in the figure, the amplitude scintillation centers on different regions and the active region transfers between the years. In 2012, the most active region is near the edge at elevation of 30° to 40° and azimuth of 220° to 260°, whereas in 2015 the most active region falls into a region with elevation of 70° to 85° and azimuth of 10° to 40°.

**Figure 12:**
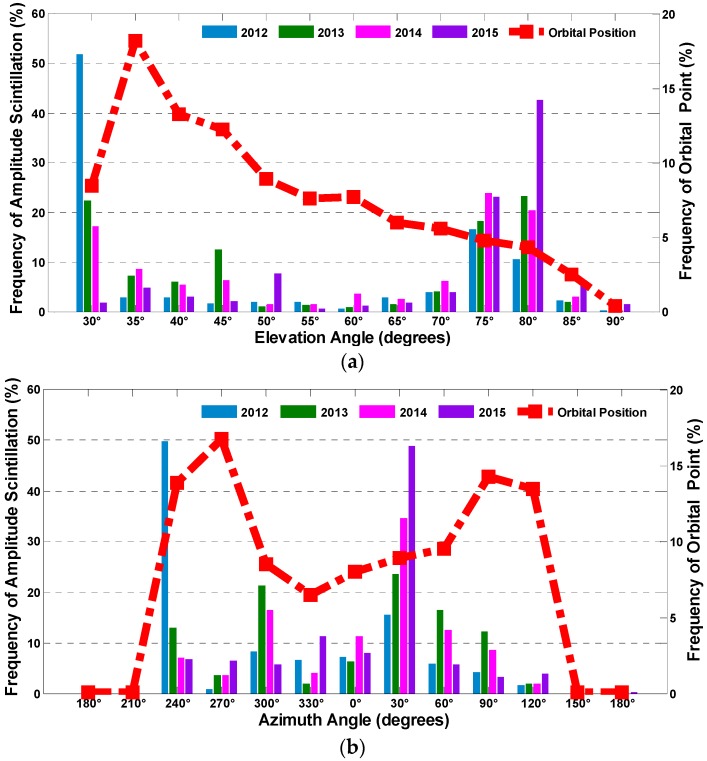
The frequency distribution of amplitude scintillation from 2012 to 2015 with: elevation (**a**); and azimuth (**b**).

Additionally, the frequency distribution of the amplitude scintillation along with the elevation and the azimuth from 2012 to 2015 is demonstrated in Figure 12 to further illustrate the relationship between the scintillation and its spatial position. It should be noted that the differences between the satellite orbits densities in different spatial regions can also contribute to the uneven distribution of scintillation in the sky, thus the percentages of satellite orbital positions falling into certain sky regions are first computed, as the red dotted lines shown in Figure 12.

In this experiment, the elevation interval varies from (30 ± 2.5)° to (90 ± 2.5)° by steps of 5°, while the azimuth varies from (0 ± 15)° to (330 ± 15)° by steps of 30°. It can be seen that most of the amplitude scintillation falls into the region of elevations around 30° and within 75° to 80°, and azimuths around 240° and 30°, which is not related to the distribution of satellite orbital positions. This result also shows that the frequency of amplitude scintillation in different spatial regions varies significantly over the analyzed period.

Comparing the height of the bars, it can be obviously seen that the frequency changes gradually as the year passes by, that is to say the distribution of the amplitude scintillation is not invariant. To be exact, for elevation, the frequency decreased gradually near 30° and increased at 75° and 80° year by year. For Azimuth, the frequency decreased at 240° and increased at 30°. These gradual changes indicate that the active regions shift between years, which is consistent with Figure 11. However, the reason for the transfer of the amplitude scintillation active area is beyond the scope of this paper. Further research should be conducted in a separate study.

**Figure 13:**
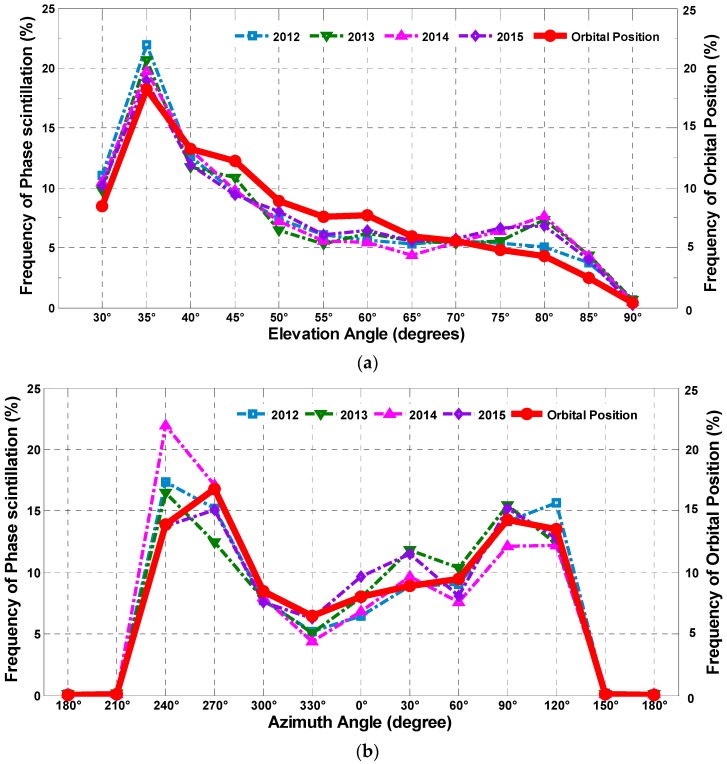
The frequency distribution of phase scintillation from 2012 to 2015 with: elevation (**a**); and azimuth (**b**).

Correspondingly, the frequency distribution of the phase scintillation along with the elevation and the azimuth from 2012 to 2015 are also separately demonstrated in Figure 13. Unlike amplitude scintillation, the active regions for phase scintillation are strongly related to the orbit position distribution. In other words, the phase scintillation is more likely to occur in the region where satellite paths are denser, such as at elevations around 35° and azimuths around 120° and 240°. In addition, it can be seen from the figure that the percentages of phase scintillation are higher than that of orbital positions near elevations of 35° and 80°, which agrees with the amplitude scintillation distributions with elevation in Figure 11a. For phase scintillation distribution along azimuth, the percentages near 240° and 30° are higher. These regions are likely to be the magnetic zenith, around which the geomagnetic field disturbance is relatively frequent and strong. This result is meaningful for exploring the contributory factors for scintillation in this region. In addition, it provides essential information for improving the positioning accuracy when GNSS is applied locally.

## 4. Concluding Remarks

In this paper, the temporal and spatial statistical characteristics of ionospheric scintillation observed at Macquarie Island have been studied with the data sets provided by SWS from year 2011 to 2015. Based on the analysis, the basic conclusions for monthly and daily scintillation have been verified, indicating that the ionospheric scintillation in sub-Antarctica region follows universal regulations. However, what makes the research more meaningful are some abnormal features of scintillation presented in the analysis based on the long-term observation. These results are of great significance for further research on global scintillation principles.

A general picture of the scintillation activities has been first investigated to demonstrate the frequency of scintillation in different months and years. The seasonal variation of amplitude scintillation is apparently found with maximum in spring equinox and autumn equinox, and minimum in summer and winter. By contrast, this is not applicable with the phase scintillation. Instead, the phase scintillation achieves peaks in summer and winter in most years. Additionally, it can be concluded that the phase scintillation is drastically more frequent than the amplitude scintillation, which is consistent with many previous studies. Furthermore, it is found that the scintillation of weak levels takes up the largest part in every month, and the stronger scintillation is likely to appear when amplitude and phase scintillation occur at the same moment.

To further analyze the influence of solar activity on scintillation, the monthly and daily occurrence rates of scintillation are calculated and the correlation with *SSN* is computed. It can be concluded that the amplitude scintillation correlates positively with the monthly smoothed *SSN*. The peak value occurs in April 2014, which is exactly the predicted official maximum for solar activity cycle 24. Meanwhile, the ratio of phase scintillation does not show apparent dependence on *SSN*. On the contrary, the phase scintillation is more frequent in 2015. More importantly, it can be seen that there is an obviously gradual increase in September, October, November and December among different years, which is really opposite with the regularity of solar activity. Furthermore, the correlation coefficients between daily scintillation rates and *SSN* are calculated, showing that there is little linear relationship between the variation of solar activity and the daily occurrence rates, for both amplitude and phase scintillation. We suppose that the effect of solar activity on scintillation is more reflected as an indirect cumulative effect, especially for amplitude scintillation. For phase scintillation, solar activity is likely to have less impact on it.

Another factor modifying scintillation researched in the work is the geomagnetic activity. In this research, the influences of this disturbance on the monthly and daily scintillation occurrences are also studied based on the data selected from the specially chosen eight months from 2012 to 2015. The correlation coefficients are calculated for amplitude and phase scintillation, respectively. Compared with *SSN*, the geomagnetic field index presents a much higher correlation with the phase scintillation, indicating that scintillation (especially daily scintillation) has a stronger dependence on *Ap* and *Ae*. Unlike phase scintillation, the correlation between *Ap*, *Ae* and amplitude scintillation remains low and it decreases gradually year by year, which deserves a further research.

The geomagnetic field horizontal component *H* measured at Macquarie Island is employed to further demonstrate the relevance between the geomagnetic field activity and the scintillation. The results manifest that there exist a high correlation and an apparent regularity between the standard deviation of *H* and the phase scintillation. By comparing the correlation coefficients, it can be concluded that the phase scintillation depends more on the local geomagnetic field component *H*. The result is helpful for global scintillation predicting, monitoring, mapping and modeling.

Another important point in this paper is the spatial distribution characteristics of scintillation. The frequency of amplitude and phase scintillation appeared in different regions in the sky is investigated. For amplitude scintillation, the most active region changes gradually yearly. However, the frequency distribution for phase scintillation stays stable and mainly focuses on two regions during the researched time span. The result is vital and has guidance significance for GNSS application near this area.

Further studies will concentrate on the theoretical mechanism of the relationship between phase scintillation and the geomagnetic activity in the sub-Antarctica region, based on the conclusions drawn in this work. Additionally, studies of ionospheric scintillation and its spatial distributions based on a longer time series and a larger number of sites can also be considered as one of the key issues for deep discussion.

## Figures and Tables

**Table 1:** The number of days with scintillation data available.

Year	2011	2012	2013	2014	2015
Months	Days	Days	Days	Days	Days
January	Null	31	Null	31	31
February	Null	29	10	28	28
March	Null	31	31	31	31
April	13	30	30	29	30
May	30	19	31	31	31
June	28	30	30	19	11
July	29	31	31	31	Null
August	30	27	31	28	Null
September	25	30	30	30	21
October	Null	30	31	31	31
November	Null	30	30	30	30
December	30	17	30	30	30
Total	185	335	315	349	274
Percentage	50.68%	91.51%	86.30%	95.89%	75.07%

**Table 2:** The parameters provided by SWS GPS ISMs observation files.

Parameters Contained in Files	Instruction
Time	The data record universal time, in decimal hours
PRN	Pseudo-Random Number of the GPS satellite
Azimuth angle	Azimuth angle of the GPS satellite
Elevation angle	Elevation angle of the GPS satellite
L1 Carrier to Noise Ratio	*CN*0 of the signal measured on the *L*1 frequency
Amplitude Scintillation Index *S*_4_	The raw “*S*_4_” amplitude scintillation index
*S*_4_ Index Correction(*S*_4_Corr)	The estimated error to raw *S*_4_ due to internal receiver noise
FINAL *S*_4_ Index (Final_*S*_4_)	Final_*S*_4_ = *S*_4_ − *S*_4_Corr
Phase Scintillation Index (Sigma60)	The raw observed phase scintillation, calculated by the standard deviation of the carrier phase over 1 min

**Table 3:** The definition of scintillation intensity.

*S*_4_ or *σ_φ_* Index	Scintillation Intensity
<0.2	no scintillation
0.2~0.4	weak
0.4~0.6	moderate
≥0.6	strong

**Table 4:** The months selected to evaluate the impact of solar activities on daily scintillation.

Year	2012	2013	2014	2015
Amplitude Scintillation	April	November	April	April
Phase Scintillation	July	June	October	October
